# Association of county level poverty with mortality from primary liver cancers

**DOI:** 10.1002/cam4.7463

**Published:** 2024-08-03

**Authors:** Matthew Ledenko, Tushar Patel

**Affiliations:** ^1^ Division of Gastroenterology and Hepatology, Department of Transplantation Mayo Clinic Florida Jacksonville Florida USA

**Keywords:** geospatial analyses, health disparities, hepatobiliary cancer, social determinants of health, social vulnerability

## Abstract

**Background:**

The highly variable occurrence of primary liver cancers across the United States emphasize the relevance of location‐based factors. Social determinants such as income, educational attainment, housing, and other factors may contribute to regional variations in outcomes. To evaluate their impact, this study identified and analyzed clusters of high mortality from primary liver cancers and the association of location‐based determinants with mortality across the contiguous United States.

**Methods:**

A geospatial analysis of age‐adjusted incidence and standardized mortality rates from primary liver cancers from 2000 to 2020 was performed. Local indicators of spatial association identified hot‐spots, clusters of counties with significantly higher mortality. Temporal analysis of locations with persistent poverty, defined as high (>20%) poverty for at least 30 years, was performed. Social determinants were analyzed individually or globally using composite measures such as the social vulnerability index or social deprivation index. Disparities in county level social determinants between hot‐spots and non‐hot‐spots were analyzed by univariate and multivariate logistic regression.

**Results:**

There are distinct clusters of liver cancer incidence and mortality, with hotspots in east Texas and Louisiana. The percentage of people living below the poverty line or Hispanics had a significantly higher odds ratio for being in the top quintile for mortality rates in comparison to other quintiles and were highly connected with mortality rates. Current and persistent poverty were both associated with an evolution from non‐hotspots to new hotspots of mortality. Hotspots were predominantly associated with locations with significant levels of socioeconomic vulnerability or deprivation.

**Conclusions:**

Poverty at a county level is associated with mortality from primary liver cancer and clusters of higher mortality. These findings emphasize the importance of addressing poverty and related socio‐economic determinants as modifiable factors in public health policies and interventions aimed at reducing mortality from primary liver cancers.

## Graphical abstract

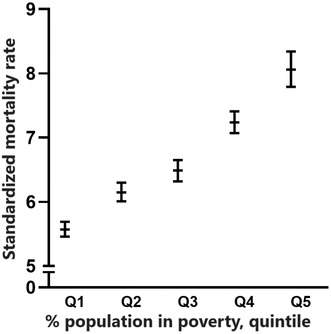

## 1 INTRODUCTION

Primary liver cancers (PLC) are a major cause of cancer‐related burden globally. There are several distinct histological types of PLC with different cells of origin, genetics and risk factors. The major types are hepatocellular carcinoma and cholangiocarcinoma. The development of these cancers involves multiple factors, often involving chronic inflammation from chronic hepatitis B or C infections, toxins, or metabolic disorders that lead to cirrhosis.

Public health measures and preventative measures that target specific risk factors, such as chronic viral hepatitis, have decreased liver cancer mortality. Despite this, their incidence is projected to increase by 55% by 2040.^1^ Understanding the modifiable factors that contribute to these trends will form the basis for efforts to reverse them.

There are significant socio‐economic and racial/ethnic disparities in the incidence and outcomes of liver cancer.^2^, ^3^ Notably, there are also significant geographical differences in cancer incidence and mortality across the United States.^4^, ^5^, ^6^ These disparities imply that local and geospatially diverse factors play an etiological or contributing role in cancer risk in addition to other established risk factors. Individual genetic predisposition and disease susceptibility, environmental exposures, or population‐based determinants could all contribute to the observed geographic variations.^7^, ^8^, ^9^ The impacts of social determinants such as income, access to food, education, insurance, and transportation, or neighborhood deprivation in determining the risk of developing or dying from cancer are increasingly being recognized.^10^, ^11^ Composite measures of several determinants, such as the social vulnerability or social deprivation indices, have also been identified as potential risk prediction tools for cancer mortality.^12^, ^13^

While location‐based differences in social determinants have been reported for liver cancer, they remain inadequately characterized and have focused on regional analyses.^14^, ^15^ To evaluate these within a broader context, this study conducted a geospatial analysis of PLC across the contiguous United States to identify clusters of disproportionately high liver cancer mortality (hot‐spots) and their relationship with location‐based factors. This approach combines detailed county‐level cancer data with emerging techniques for geospatial analysis that have been used to analyze clusters with factors such as access to healthcare or diagnosis stage.^16^, ^17^ Disparities in household income and poverty were associated with hot‐spots of mortality, and high levels of either current or persistent poverty identified locations at greatest risk of becoming new hotspots.

## 2 METHODS

### 2.1 Ethical approval

Publicly accessible data devoid of personally identifiable or protected health information was used for this study. According to the Mayo Clinic Institutional Review Board, review and ethical approval were not required for this type of study involving human subjects. The study was conducted in accordance with ethical principles described in the Declaration of Helsinki of 1975 (Seventh Revision, 2013).

### 2.2 Demographics

The people living in the contiguous states of the U.S. made up the study population. County‐level social, demographic, economic, and housing data were obtained from the US Census Bureau's American Community Survey (ACS), Decennial Census, the Food Environment Atlas by the United States Department of Agriculture, and other sources from 2010 up to 2020 as described in Table S1. Data used in analyses were cross‐sectional or 5‐year averages.

### 2.3 Cancer mortality and incidence

County‐level data on total population, deaths, and standardized mortality rates (SMR) from PLC were obtained from the Centers for Disease Control and Prevention (CDC), Wide‐ranging Online Data for Epidemiologic Research, using the International Classification of Diseases‐10 site code C22.0. Data on age‐adjusted incidence rates (AAIR) of PLC were obtained from the United States Cancer Statistics (USCS) Public Use Databases from the CDC. Mortality data were suppressed if fewer than 10 cases, and incidence data if fewer than 16 cases were reported per county. The identification, screening and selection of counties for analysis are outlined in Figure S1.

### 2.4 Geospatial analysis

The cancer data were matched to their respective county polygons within the 2022 TIGER/line shapefile from the US Census Bureau. Since redistricting in Connecticut in 2021 affected county borders and identities, the shapefiles used did not include those counties. Global spatial association was analyzed by performing a regression analysis of the rate for a county against that of its neighbors to derive a global Moran's I statistic (GMI). The GMI indicates the degree to which a spatial pattern is clustered (+1), dispersed (−1) or random (0). Geospatial association was analyzed using the Local Indicator of Spatial Autocorrelation (LISA) to measure whether the rate for a given county was closer to that of an adjacent county or the average across the US. A local Moran's I‐statistic was generated per county, with positive values indicating similarity and negative values indicating dispersion. Counties were grouped into categories and described with the first attribute (high or low) indicating the county's rate and the second attribute (high or low) indicating the neighboring county's rate in comparison to the nation. A non‐significant designation indicated either the absence of significant spatial correlation or lack of contiguity. Thus, an HH (high‐high) county indicates a hot spot where a county with high rates is surrounded by counties with similarly high rates. The statistical significance was tested using a Monte Carlo approach with 999 permutations. Mapping and visualizations were generated using ArcGIS Pro version 3.0.3 (Environmental Systems Research Institute, Redlands, CA). Geospatial analysis was conducted using GeoDa version 1.20 (CSDS, Chicago, IL) and using Queen's case adjacency rules to determine the contiguity of counties.

### 2.5 Poverty

Poverty is described as having a total income that is less than a federally set threshold that is adjusted annually. Below this threshold, a family of a certain size doesn't have enough money to buy basic needs like food, shelter, clothes, and other necessities.^18^ County‐level poverty rates for 1980, 1990, 2000 and 2010 were obtained from U.S. Census Bureau Small Area Income and Poverty Estimates. High poverty was defined as ≥20% of population living in poverty per the U.S. Census Bureau.^19^ Counties were grouped by their population living in poverty as (a) never in poverty, in which high poverty was not experienced, (b) intermittent high, in which high poverty was experienced at least once or (c) persistent poverty in which a county experienced high poverty at every timepoint between 1980 and 2010.

### 2.6 Composite indices

Composite measures that incorporate poverty measures with other socio‐demographic and economic factors have been used in ecological studies of cancer incidence and to measure socio‐economic differences in health outcomes.^12^, ^20^ The social vulnerability index (SVI) encompasses several factors in the following domains: socioeconomic, household composition and disability, minority status, and language, and housing type and transportation. County‐level SVI data was obtained from the CDC/Agency for Toxic Substances and Disease Registry for 2010 and 2020. Similarly, the social deprivation index (SDI) is derived from seven variables collected in the ACS, namely the percentage of people living in poverty, rental housing units, overcrowded housing, households with single parent, no car, unemployed adults with less than 12 years of education, or under 65 years. The SDI was obtained from the Robert Graham Center for 2012 and 2019.^21^ Both indices are reported as a percentile ranking, with 100 representing the most vulnerable or deprived counties.

### 2.7 Statistical analysis

The statistical significance of global spatial association was tested using a Monte Carlo approach with 999 permutations. Comparison of factors associated with hot‐spots or cold‐spots were assessed using Welch two‐sample, two‐sided *t*‐tests. Multivariate logistic regression analysis was performed for factors that were significantly different between HH and LL counties (*p* < 0.05) in the fifth quantile compared with other quantiles for SMR and AAIR. Results are reported as odds ratios (ORs) and 95% CIs. Analyses were performed using BlueSky version 7.4 (BlueSky Statistics LLC, Chicago, IL).

All authors had access to the study data and reviewed and approved the final manuscript.

## 3 RESULTS

### 3.1 Clusters of incidence of primary liver cancer

**FIGURE 1:**
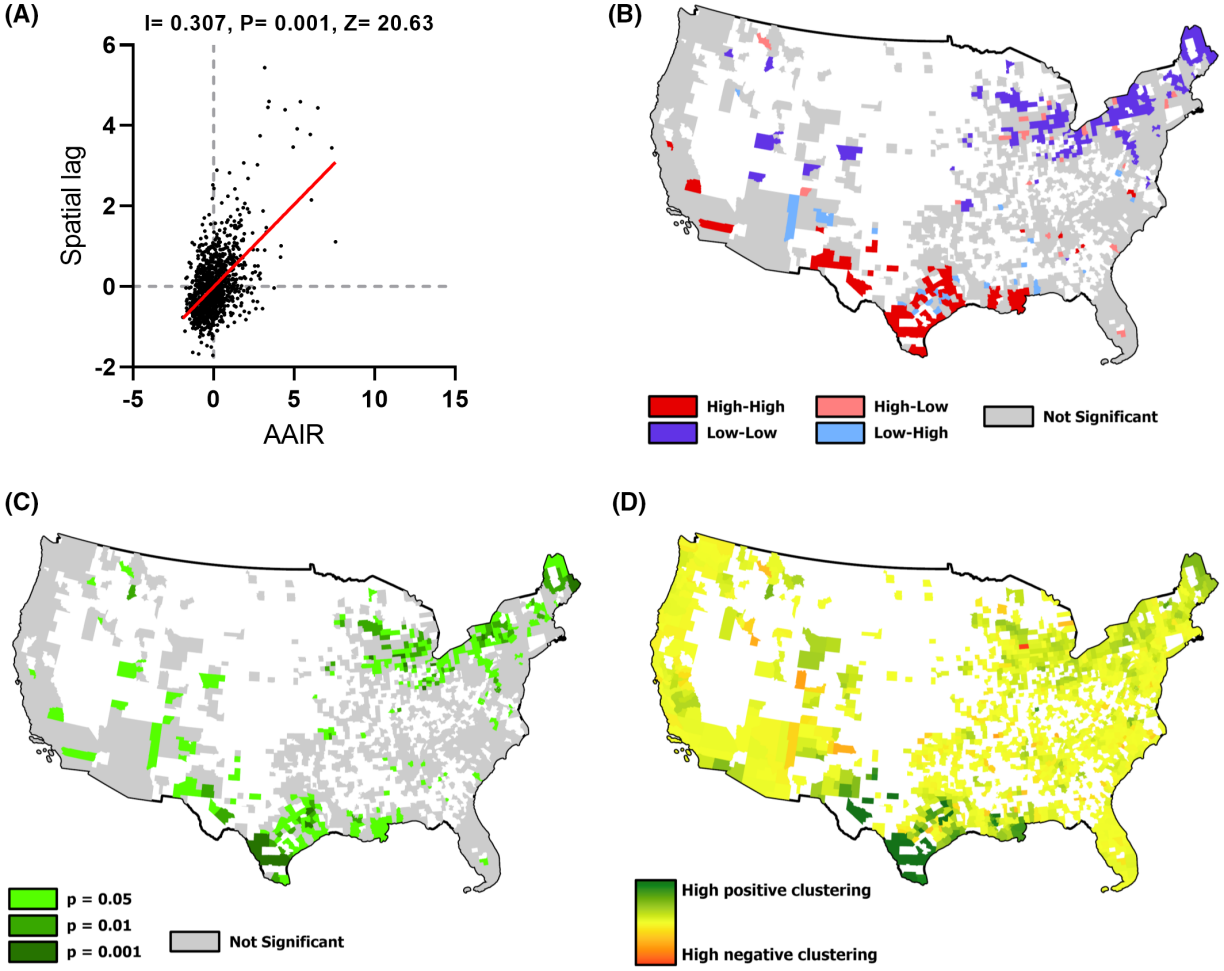
Geospatial analysis of the incidence of primary liver cancer. (A) Global spatial association analysis of age‐adjusted incidence rate (AAIR) with global Moran's I, *p* value, and *Z*‐statistic indicated. (B–D) Geospatial association showing (B) significant disease clusters in color, and non‐significant clusters in gray, (C) corresponding *p* values and (D) geographic clustering.

Geographic regions with a higher incidence of liver cancer could benefit from targeted measures to increase awareness. To identify these regions, we analyzed the AAIR for liver cancer between 2015 and 2019 across 1445 counties for which data was available. Of these, the data for one county (Union County, Florida) reported an inexplicably high pan‐cancer incidence and was excluded from analyses as a potential outlier. Amongst the rest, the AAIR for liver cancer varied considerably and ranged from 3.3 to 31.1 per 100,000 of the population. Geospatial analysis identified a GMI for AAIR of 0.307 (*p* = 0.001), indicating highly significant global spatial clustering (Figure 1). LISA analysis identified several highly significant clusters of which 87 counties were hot spots with increased incidence and 156 were cold‐spots. Texas is recognized as having the highest rates of hepatocellular cancer (HCC) in the U.S.^22^ Consistent with this, many hot spots were identified within coastal regions and East Texas, with a high level of spatial association of hot spots across the state. Hot spots were also noted in Louisiana and California. Cold spots occurred predominantly in northern or northeastern states such as Maine, New York, and Ohio, with the greatest spatial association noted in Maine. Clusters with a higher incidence of liver cancer identify geographic regions that could benefit from targeted measures to increase awareness.

### 3.2 Geospatial distribution of deaths from liver cancer

**FIGURE 2:**
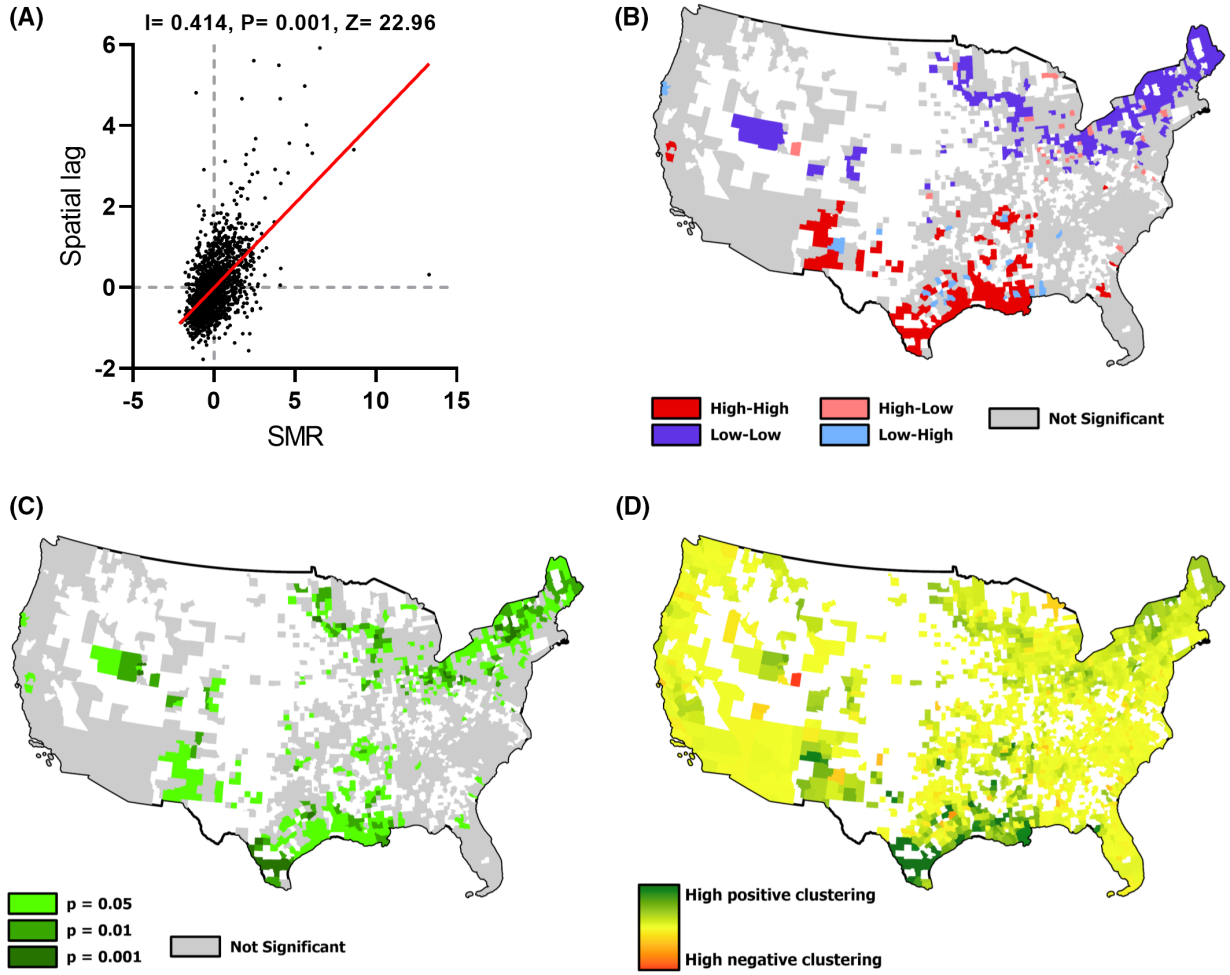
Geospatial analysis of mortality from primary liver cancer. (A) Global spatial association analysis of standardized mortality rate (SMR) with global Moran's I, *p* value, and *Z*‐statistic indicated. Local indicators of spatial association analysis identified (B–D) Geospatial association showing (B) significant clusters of mortality in color, and non‐significant clusters in gray, (C) corresponding *p* values and (D) geographic clustering.

A similar geospatial analysis was performed for liver cancer mortality between 2010 and 2020 across the US. A total of 259,651 deaths were reported from 1750 counties for which data was available. The GMI for SMR for liver cancer was 0.414 (*p* = 0.001), and LISA analysis identified highly significant clusters with 159 hot‐spots and 215 cold‐spots of SMR (Figure 2). There was a strong local spatial association for mortality in counties in coastal areas along the Gulf of Mexico and in South Texas. As with incidence, hot spots of mortality occurred in South Texas, but a greater number of hot spots were identified in Louisiana and Arkansas. Cold spots were similarly located in the northern states, with clusters in Minnesota, Ohio, and Pennsylvania. The minor differences between clusters of mortality and incidence may reflect data availability since incidence data was suppressed in low prevalence regions and was not as widely available as the county‐level mortality data. Similar results were obtained on a geospatial analysis of SMR between 2000 and 2010, but hot‐spots were identified during this earlier decade (Figure S2).

### 3.3 Location‐based determinants of clusters

County characteristics, demographic, racial, and ethnic composition, and social determinants were compared between hotspots and non‐hotspots of liver cancer incidence and mortality. Spatially isolated counties without any neighbors were excluded from analysis (38 counties for incidence and 32 counties for mortality). Analysis was performed on AAIR within 1406 counties from 2015 to 2019 and on SMR in 1718 counties from 2010 to 2020. Significant differences were found between hot‐spots and non‐hot‐spots of either incidence or mortality in population density and the percent of population in poverty, Hispanics, aged >65, or aged 25+ with a bachelor's degree (Table 1).

**TABLE 1:** County‐level variables across clusters of liver cancer incidence and mortality.

	Variable	Incidence			Mortality		
		Non‐hot‐spots mean (*n* = 1319)	Hot‐spots mean (*n* = 87)	*p*‐value	Non‐hot‐spots mean (*n* = 1559)	Hot‐spots mean (*n* = 159)	*p*‐value
County characteristics	Total pop.	203,798	205,128	0.9	183,720	140,468	0.2
	Pop. density	548.72	214.75	0.0002	493.12	175.97	<0.0001
	Percentage of houses in rural areas	43.37	42.35	0.8	47.65	50.41	0.3
Demographic	Males per 100 females	97.97	102.60	0.002	98.62	100.00	0.2
	% pop. >65 years of age	17.70	15.76	<0.0001	17.76	16.52	0.0002
Racial and Ethnic	Ratio of black to white	0.19	0.27	0.043	0.19	0.33	<0.0001
	% pop. that is Hispanic	9.84	35.29	<0.0001	9.42	20.74	<0.0001
	% pop. that is Asian	2.21	2.65	0.019	2.00	1.60	0.1
Social determinants	% pop. in poverty	12.88	16.50	<0.0001	12.87	17.51	<0.0001
	% households without car	6.31	5.02	0.0008	6.30	6.60	0.2
	% pop. >25 years of age with bachelors	15.76	13.05	<0.0001	15.41	12.58	<0.0001
	% pop. >25 years of age with high school equivalency	31.95	31.75	0.8	32.56	33.93	0.025
	% single parent households	6.35	5.80	0.8	6.10	7.76	<0.0001

**FIGURE 3:**
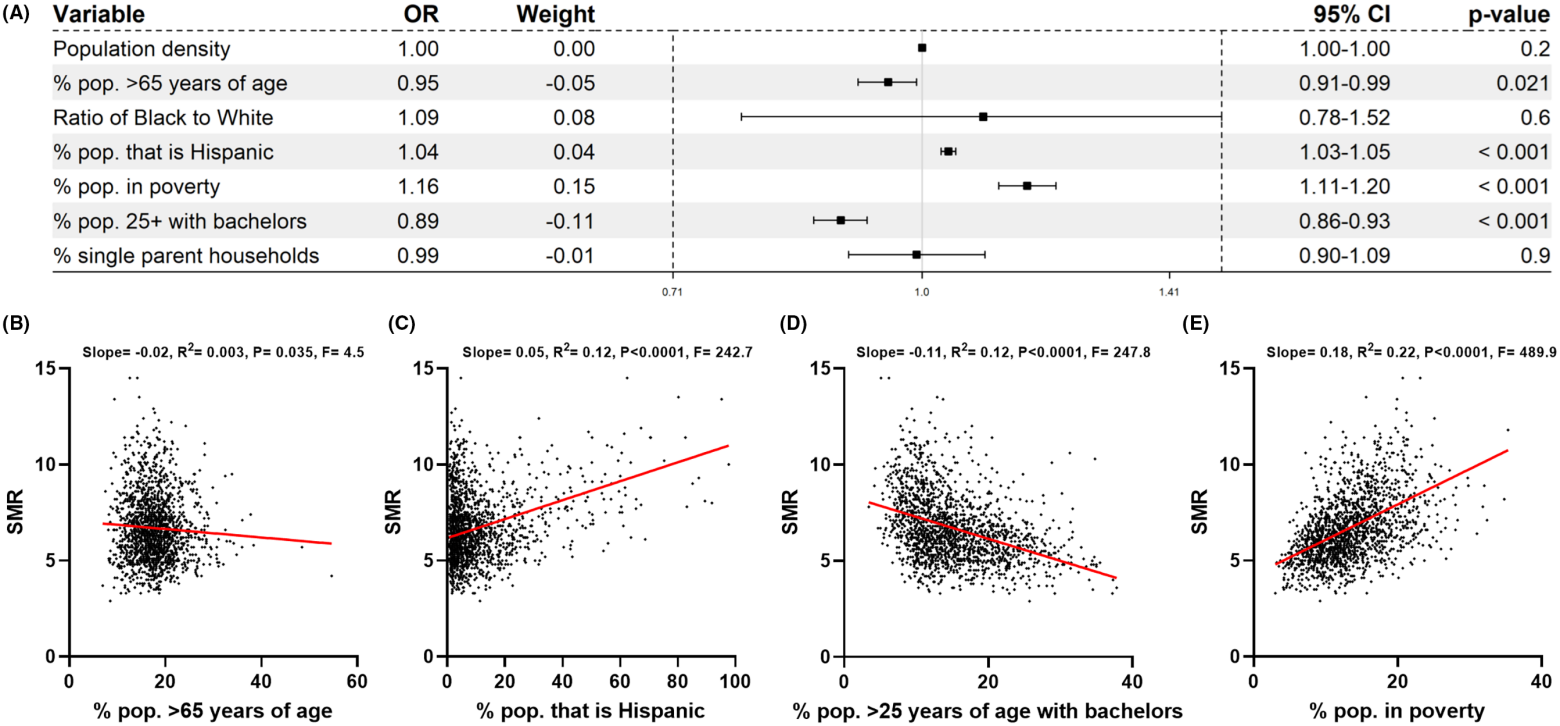
County level determinants of liver cancer mortality. (A) Multivariate logistic regression analysis, with odds ratio (OR) for being within the top quintile versus the bottom four quintiles of the standardized mortality rate (SMR) for liver cancer in 2010–2020. Correlation of SMR with the percent of the population (B) >65 years old (C) of Hispanic origin (D) >25 years of age with a bachelor's degree and (E) in poverty for counties in the US. The slope, *R*‐squared, *p*‐value, and *F*‐statistic are indicated for each plot.

A multiple logistic regression analysis was performed on variables with significance level *p* < 0.001 on univariate analysis, and the odds ratio (OR) determined for an SMR within the fifth (top) quintile to other quintiles (Figure 3). The percentage of population that were Hispanics or living below the poverty line had an OR >1, whereas the percentage of population aged 25 and older with a bachelor's degree, or aged 65 years or more had an OR <1. Amongst these variables, the percent of population in poverty within a county showed the greatest correlation with county level SMR for liver cancer across all 1718 counties. However, the percentage of population aged 25 and older with a bachelor's degree, or aged 65 years or more did not correlate well with mortality from liver cancer.

### 3.4 Temporal evolution of mortality risk

We sought to identify location‐based determinants of progression to worsening mortality. Geospatial analysis was performed to identify hot‐spots of mortality in each of two consecutive decades, 2000–2010 (D1) and 2010–2020 (D2). A total of 1219 counties were identified as non‐hot‐spots for liver cancer mortality in the first decade. Amongst these 1168 remained non‐hot‐spots whereas 51 became new hot‐spots in the second decade. An analysis of county‐level demographics, race, ethnicity, and income were analyzed between those non‐hotspots that became hot‐spots, and those that remained non‐hotspots (Table 2). Several income related factors and the proportion of population of any minority during the first decade were significantly different in counties that became new hot‐spots compared with those that did not. Several health or health‐care related factors varied between new hot‐spots and those that remained non‐hot‐spots (Table S2). County level income related factors were the most important determinants of progression from a non‐hot‐spot to a hot‐spot region. Moreover, disparities in these factors were present during both decades between non‐hot‐spots and new hot‐spots.

**TABLE 2:** County‐level features of non‐hotspots in decade 1 (2000–2010) that either remained non‐hotspots or became hotspots in decade 2 (2010–2020).

	Variable	Remained non hot‐spots (*n* = 1168)			Became new hot‐spots (*n* = 51)			Non‐hot‐spots versus new hot‐spots decade 1 *p*‐value
		Decade 1 mean	Decade 2 mean	*p*‐value	Decade 1 mean	Decade 2 mean	*p*‐value	
County characteristics	Total pop.	211,785	228,301	0.4	133,379	147,099	0.8	0.016
	Pop. density	509.6	619.42	0.4	168.2	175.98	0.9	0.0002
	County size (miles^2^)	971.25		—	1106.01		—	0.4
	Percentage of houses in rural areas	38.34	39.62	0.4	39.07	41.23	0.6	0.8
Demographics	Males per 100 females	97.63	98.04	0.1	97.62	98.27	0.7	0.9
	Median age (years)	38.58	40.34	<0.0001	36.22	37.34	0.2	0.0003
	% pop. >65 years of age	14.15	17.35	<0.0001	13.35	16.29	<0.0001	0.07
Race and ethnicity	% pop. that is of any minority	22.67	25.42	0.0002	39.63	42.67	0.4	<0.0001
	Ratio of black to white	0.17	0.20	0.073	0.30	0.34	0.6	0.009
	% pop. that is Hispanic	8.28	10.20	<0.0001	16.03	19.28	0.4	0.012
	% pop. that is Asian	1.81	2.39	<0.0001	1.80	2.16	0.6	0.9
Income	% pop. poverty	15.71	12.46	<0.0001	19.87	16.53	0.0003	<0.0001
	% pop. unemployed	8.04	5.34	<0.0001	7.89	6.35	0.0009	0.6
	Per capita income (U.S. dollars)	24,566	30,431	<0.0001	21,773	26,524	<0.0001	0.0002
	Median household income (U.S. dollars)	48,659	58,394	<0.0001	41,334	49,990	0.0003	<0.0001
	% pop. without health insurance	20.45	12.39	<0.0001	26.70	16.36	<0.0001	<0.0001
	% pop. using the supplemental nutrition assistance program	15.58	13.22	<0.0001	18.15	16.58	0.018	<0.0001
	% households without car	6.70	6.49	0.2	7.09	6.81	0.5	0.2
Education	% pop. >25 years of age with bachelors	6.86	16.37	<0.0001	5.23	13.89	<0.0001	0.0004
	% pop. >25 years of age with high school equivalency	33.26	31.32	<0.0001	32.91	32.22	0.6	0.7
Housing	Household size (persons)	2.51	2.52	0.3	2.60	2.62	0.6	0.023
	Family size (persons)	3.04	3.07	0.034	3.16	3.26	0.037	0.001
	Single parent households	10.94	6.19	<0.0001	13.20	7.67	<0.0001	<0.0001
	% households that are mobile homes	10.51	9.52	0.003	15.17	15.15	0.9	0.0002
	% households that are group homes	3.04	3.01	0.8	3.33	3.40	0.9	0.6
	% households with more people than rooms	3.11	2.24	<0.0001	3.15	3.04	0.8	<0.0001
Composite metrics	Social vulnerability index	51.22	51.94	0.5	0.77	80.00	0.3	<0.0001
	Social deprivation index	41.95	46.50	<0.0001	63.39	72.88	0.007	<0.0001
	Food environment index	7.72		—	6.83		—	<0.0001

Abbreviation: Pop., population.

**FIGURE 4:**
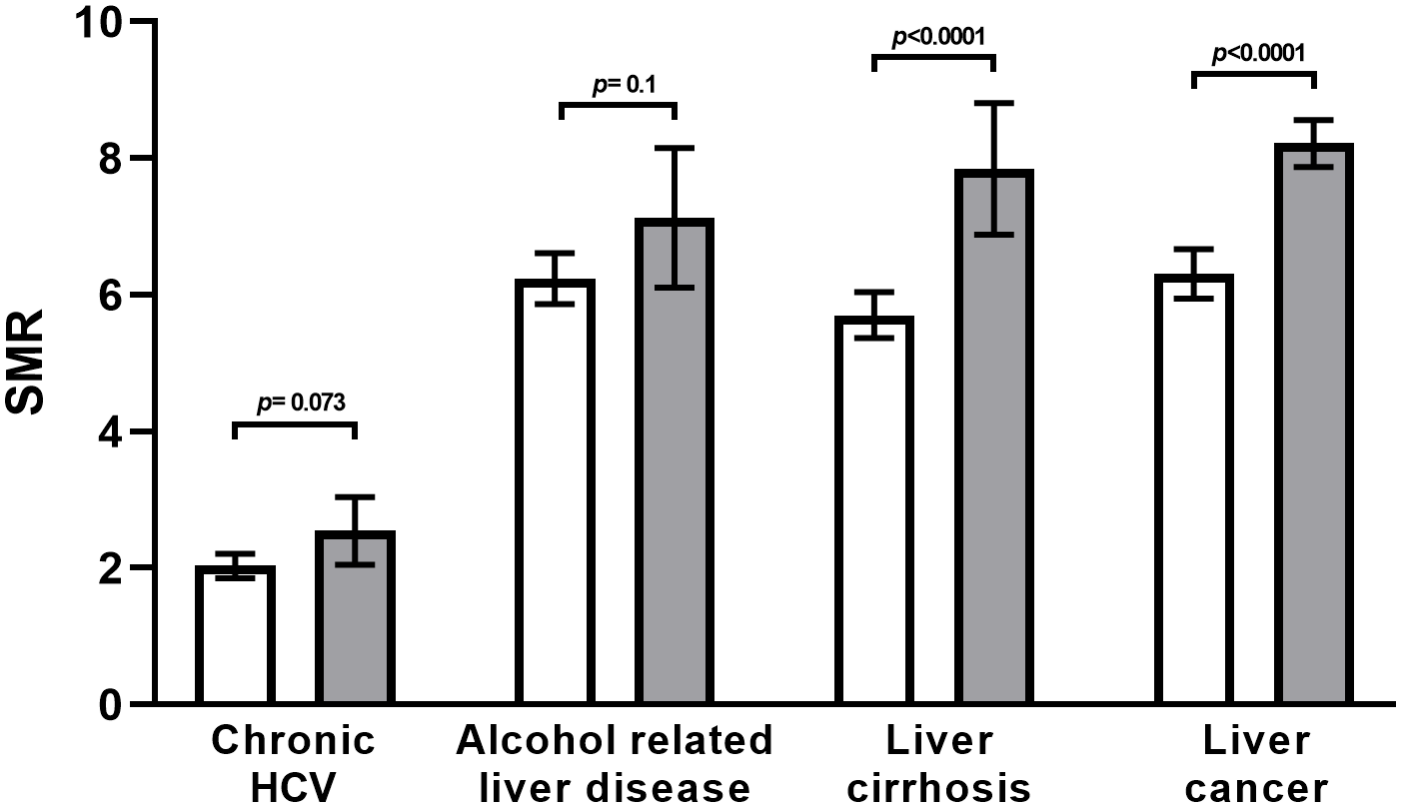
Disease related mortality in new hot‐spots and persistent non‐hot‐spots of liver cancer. Amongst 1219 counties that were non‐hot‐spots for liver cancer mortality in 2000–2010, 51 became new hot‐spots (gray bars) whereas 1168 remained non‐hot‐spots (white bars) in the subsequent decade. Disease‐related standardized mortality rates (SMR) from 2010 to 2020 are shown with error bars representing 95% confidence intervals.

Most cases of liver cancer arise in the context of liver cirrhosis. Mortality from liver cancer and liver cirrhosis were both significantly higher in new hot‐spots compared with persistent non‐hot‐spots whereas mortality from alcoholic liver disease or hepatitis C was not significantly different (Figure 4).

### 3.5 Impact of poverty

**FIGURE 5:**
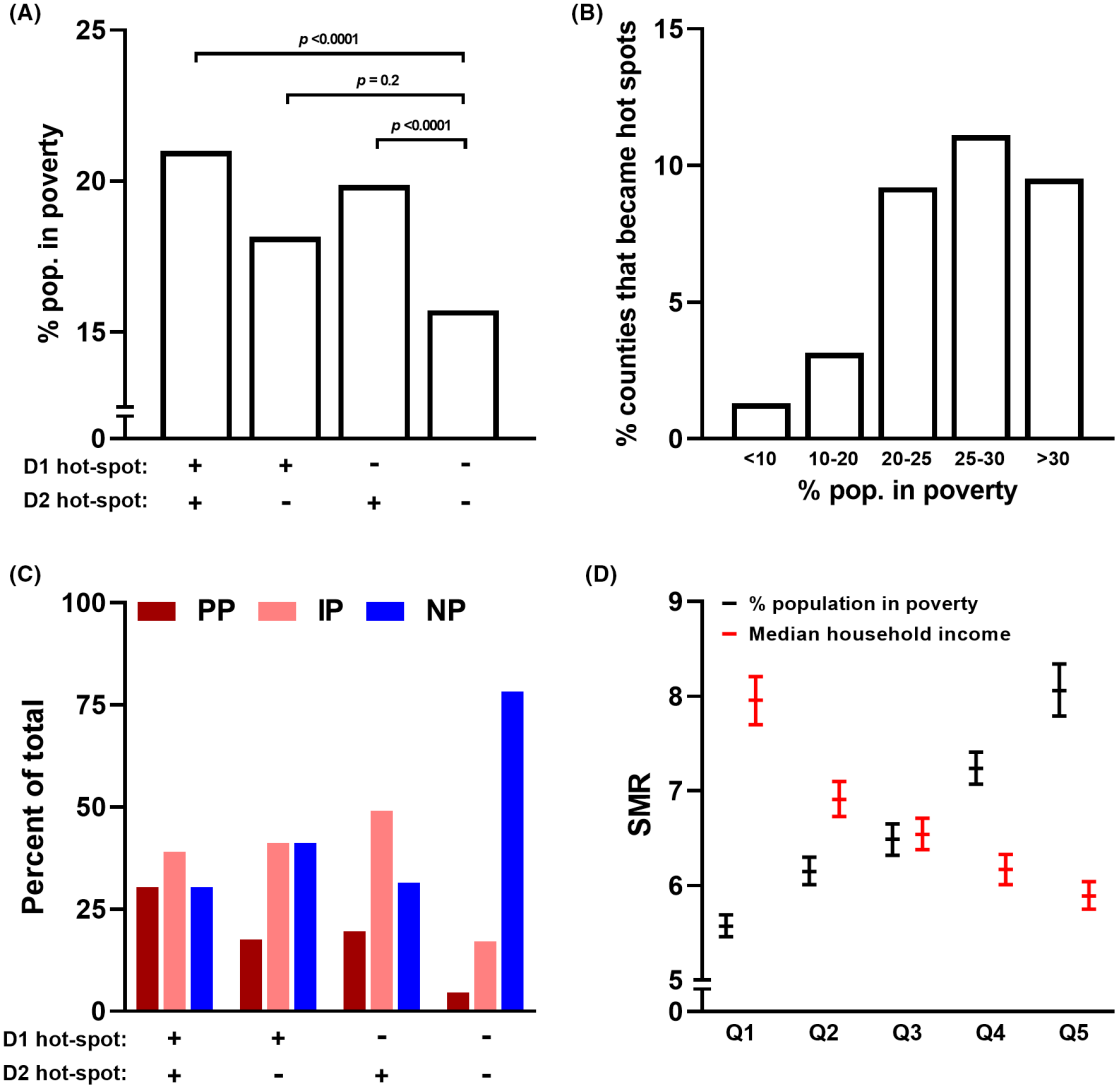
Association of poverty and liver cancer mortality. (A) The average percentage of population in poverty in 2010 is shown for groups of counties based on their identification as hot‐spots during either the first (D1) or second (D2) decades. (B) The percentage of population in poverty during 2010 was assessed for counties that were non hot‐spots in 2000–2010 but became hot‐spots in 2010–2020. (C) The percentage of counties with high poverty (poverty in >20% of county population) at four (persistent poverty, PP), 1–3 (intermittent poverty, IP) or no (never in poverty, NP) time points in 1980, 1990, 2000, and 2010 is shown for groups of counties based on their identification as hot‐spots during either the first or second decades. PP counties are shown in red, IP in pink, and NP in blue. (D) The average standardized mortality rates (SMR) in counties grouped by quintiles of their median household income (red) or proportion of population in poverty (black) in 2010–2020. Data represents means and 95% confidence intervals.

The distribution of poverty was associated with counties that subsequently became new hot‐spots. The proportions of counties with current poverty in non‐hot‐spots that transitioned to hot‐spots during the second decade were remarkably similar to those in counties that were already hot‐spots, and were also significantly different from those that were non‐hot‐spots in either decade (Figure 5A). Amongst counties with fewer than 10% of the population in poverty in 2000–2010, only 1.3% became new hot‐spots, whereas amongst counties with more than 25% poverty, 9.5%–11.1% of counties became new hot‐spots (Figure 5B).

Similarly, a history of ongoing, persistent poverty was also associated with progression from a non‐hot‐spot to a hot‐spot. The proportions of counties with persistent poverty in non‐hot‐spots that transitioned to hot‐spots during the second decade were also similar to those in counties that were already hot‐spots (Figure 5C). Across all counties, the highest morality rates were observed with in the highest quintiles of poverty or low income (Figure 5D). Thus, both current and persistent poverty at a county level can identify those with higher mortality rates as well as a greater likelihood of worsening relative mortality.

Conversely, amongst counties that were identified as hot‐spots for mortality in 2000–2010, 17 became non‐hot‐spots within the next decade. Significant differences were observed in the percent of population that were unemployed or lacked health insurance in these (Table S3). While limited by the small numbers of counties showing a favorable progression, the results of these analyses are consistent with an association of mortality with socioeconomic factors related to income or access to health care.

### 3.6 Social determinants and mortality

Social determinants of health related to poverty, such as low education, household size and unemployment, can influence healthcare access and outcomes. A broader assessment of poverty related socioeconomic disadvantage within a community is appropriate since residual confounding by unmeasured variables could impact risk. Therefore, we evaluated composite indices of social vulnerability (SVI) and deprivation (SDI). At the county level, both indices are directly related to and are influenced by poverty within that county. Other factors within the SVI such as income levels, unemployment, housing conditions and educational attainment are also related with poverty. Similarly, SDI incorporates several socioeconomic variables and has been linked to poorer health outcomes.^15^, ^23^

**FIGURE 6:**
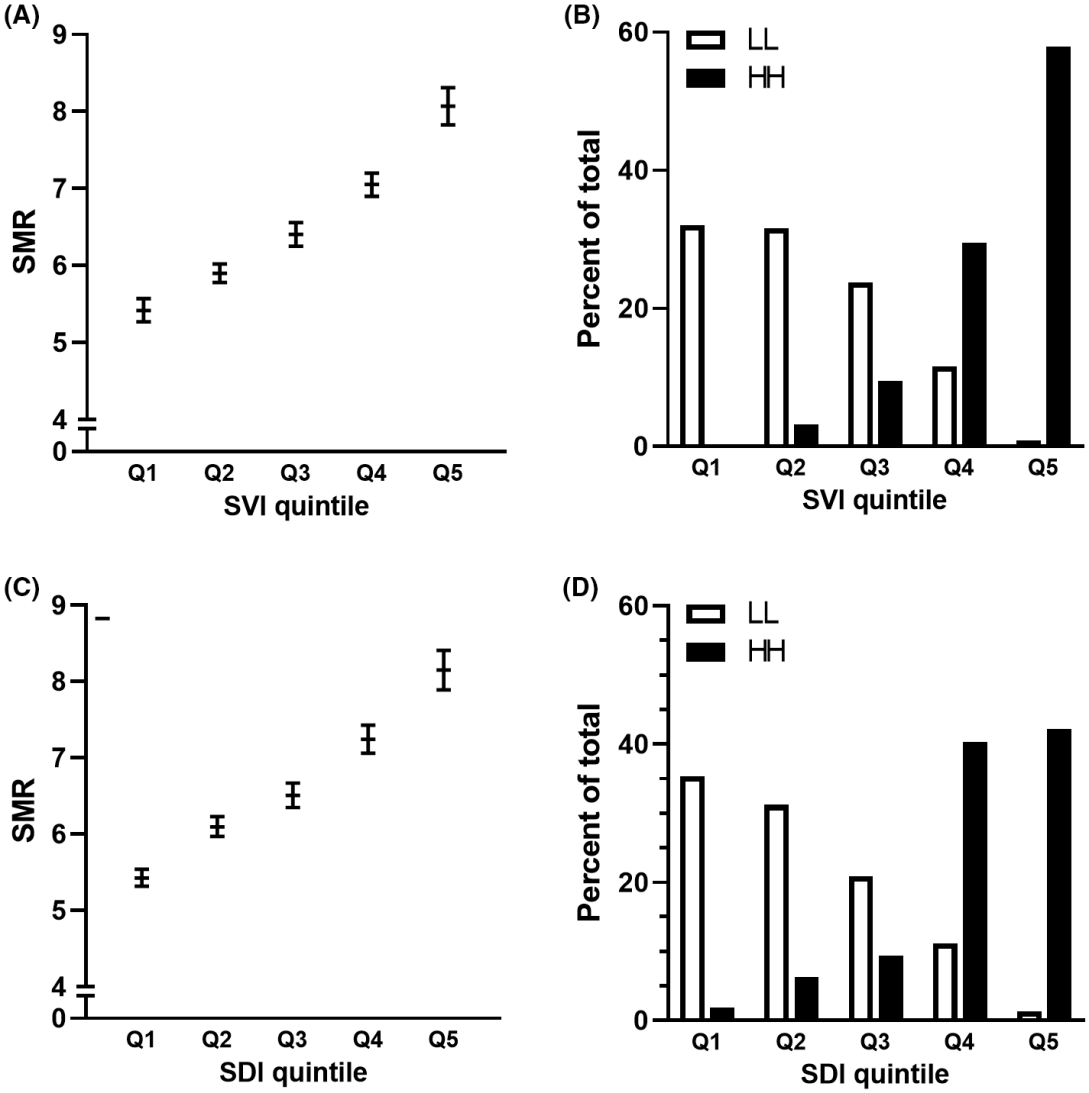
Liver cancer mortality (SMR) and social vulnerability or deprivation. (A, C). The average liver cancer SMR between 2010 and 2020 for counties within quintiles of the (A) social vulnerability index (SVI) or (C) social deprivation (SDI). Error bars represent 95% confidence intervals. (B, D). The percentage of total counties that were hot‐spots (dark bars, HH) or cold‐spots (white bars, LL) of SMR from 2010 to 2020 within each quintile of (B) SVI or (D) SDI.

The SVI showed a significant positive correlation with SMR in 2010–2020 in an analysis of 1718 counties across the US (Figure 6). The SMR increased with SVI quintiles, from 5.42 (95% CI 5.27–5.57) in the first quintile to 8.07 (95% CI: 7.83–8.31) in the fifth quintile. Based on these data, 10,616 to 15,927 fewer deaths could be projected over a decade if all counties within any one of the top four quintiles of SVI moved down to the next lower quintile. Within disease clusters the SVI was significantly higher in hot spots of liver cancer mortality than in cold spots, with 57.9% of all hot spots detected in counties that were in the top quintile of the SVI. In contrast, most cold spot counties were within Q1‐3, and only 0.9% of the identified cold spots were in Q5 for SVI.

Similar to the SVI, the mortality rate increased with SDI quintiles (Figure 6), and the mean SDI was significantly higher in hot‐spot counties (Figure S3). Strikingly, 82.4% of the mortality hot‐spots were within Q4–Q5 of SDI, whereas only 12.6% of cold‐spots were seen in Q4–Q5. Notably, both SVI and SDI were also significantly higher in new hot‐spot counties compared with persistent non‐hot‐spots. The dramatic difference in SVI and SDI distribution between clusters of high and low mortality highlights their utility for predicting communities at higher risk.

## 4 DISCUSSION

Poverty related factors can contribute to disparities in mortality in several ways. Inadequate health insurance, limited access to healthcare, or lower educational attainment can pose barriers to prevention, detection, and management of liver cancers. These factors may contribute to a higher risk of cancer seen with a lower socio‐economic status in some locations^2^, ^24^, ^25^ and amongst some racial groups.^3^, ^14^, ^26^, ^27^ The higher mortality from HCC in low‐income patients could reflect advanced‐stage disease and delays in screening, diagnosis, or the initiation of treatment.^28^, ^29^, ^30^, ^31^ Additionally, hot‐spots of mortality had higher proportions of Hispanics. Liver cancers are disproportionately higher in Hispanic person.^32^ Compared with other ethnic groups, Hispanic persons have a higher burden of hepatitis C infection, and a higher prevalence of obesity, diabetes, and metabolic syndrome.^33^, ^34^, ^35^ Moreover, susceptibility of Hispanic persons to liver cancer could be enhanced by genetic variants or environmental exposures.^36^, ^37^

The SVI and the SDI distill multidimensional socioeconomic challenges facing communities at higher risk of mortality into a composite measure of risk. While poverty measures are a key component of both indices, each provides additional distinct perspective on social inequities. These indices could be used to identify communities at high risk for targeted programs and resources aimed at reducing cancer deaths. Temporal changes in SVI or SDI over time could potentially be useful to assess the need or impact of societal policies and interventions to reduce disparities in liver cancer.

### 4.1 Limitations

There are some limitations to these analyses. Individual‐level factors (e.g., chronic viral hepatitis), screening practices, and spatial patterns of mortality and incidence finer than the county level were not examined. Aggregated data can obscure heterogeneity and lead to under‐recognition of distinct regions of high incidence or mortality within a county. There may be insufficient data from counties with smaller populations, as mortality data were suppressed for regions with small numbers of cases. The lack of data on incidence from several counties impacts the ability to identify all important clusters. Thus, the clusters identified in this study should be considered to represent a snapshot of representative counties but not a comprehensive set of all regions at low or high risk. The accuracy of coding and recording are a limitation of any data source that uses diagnosis codes. Whilst data from state or other cancer registries or the National Cancer Institute Surveillance, Epidemiology, and End Results Program may have higher accuracy in coding, their patchy coverage precluded their use for the current analysis. However, the identification of geographically localized hotspots provides a strong foundational bases and justification for conducting targeted and comprehensive epidemiological studies, as well as using high quality cancer registry data to define risks specific to these locations.

### 4.2 Conclusions

A multi‐pronged approach that strives to address individual lifestyle and risk factors as well as location‐based social, economic, and environmental conditions will be needed to reduce the burden of liver cancer in the US. Screening and public health initiatives could include culturally tailored outreach and education campaigns on liver cancer prevention, nutrition and healthy eating, promotion of physical activity or screening for viral hepatitis or liver cancer. Broader efforts could include programs to close health insurance coverage gaps that limit access to hepatitis, substance abuse and liver cancer care for the poor and uninsured, and regulation of environmental exposures. Such efforts must consider the resource constraints and obstacles related to poverty and may need to be specifically tailored towards economically challenged and vulnerable regions with high current or chronic poverty. Finally, policies to improve education, housing and economic opportunities that impact on health will be required to tackle the root causes of poverty and address social determinants to create health equity and improve outcomes for liver and other cancers.

## AUTHOR CONTRIBUTIONS

**Matthew Ledenko:** Formal analysis (equal); investigation (lead); visualization (lead); writing – original draft (supporting); writing – review and editing (supporting). **Tushar Patel:** Conceptualization (lead); funding acquisition (lead); methodology (lead); supervision (lead); writing – original draft (lead); writing – review and editing (lead).

## CONFLICT OF INTEREST STATEMENT

The authors have no relevant interests to disclose.

## PRECIS

While there are marked geographic disparities in the incidence and mortality of primary liver cancers, their causes are inadequately understood. This study finds a strong association between county‐level poverty and the liver cancer mortality rates in the United States.

## Supporting information


Data S1.

## Data Availability

All data used is publicly available from the indicated sources.
