# Supplementary material for: Association of county level poverty with mortality from primary liver cancers
Source: Cancer Med. 2024 Aug 3;13(15):e7463. doi: 10.1002/cam4.7463 (PMC11297446; doi:10.1002/cam4.7463)
Supplement: Supplementary file 1 — Data S1. [file CAM4-13-e7463-s001.docx]

**SUPPLEMENTAL DIGITAL CONTENT**

**Association of county level poverty with mortality from primary liver cancers**

Matthew Ledenko, BS, Tushar Patel, MBChB

**Figures**

Fig. S1: Selection of counties for analysis.

Fig. S2: Geospatial analysis of mortality from primary liver cancer (2000-2010).

Fig. S3: Social deprivation index within clusters of liver cancer mortality

**Tables**

Table S1: Data sources.

Table S2: Health and healthcare in counties that remained non-hot-spots or became new hot-spots.

Table S3: Features of hot-spot counties that became not hot-spots.

US counties identified via US Census Bureau 2022 TIGER/Line shapefile (n = 3,236)

**Identification**

Counties within contiguous United States

Counties excluded

(n = 126)

Counties with stable designations

Counties excluded due to altered county designations

(n = 9)

**Screening**

County level data available for analysis of :

Mortality (SMR 2010-2020) or

Incidence (AAIR 2015-2019)

Reasons excluded:

1,350 counties with SMR data not available or <10 cases

1,656 counties with AAIR data not available or <16 cases

Reason 1 (n = )

Reason 2 (n = )

Reason 3 (n = )

etc.

Counties excluded due to inexplicably high pan-cancer incidence ( n=1)

Counties within location-based clusters

(n= 1,718 for SMR )

(n= 1,406 for AAIR )

Counties with available SMR data (n = 1,750)

Counties with available AAIR data (n = 1,444)

Spatially isolated counties without neighbors

(n = 38 for AAIR, n=32 for SMR)

**Included**

**Fig. S1: Inclusion and exclusion of counties.** AAIR, age-adjusted incidence rate, SMR: standardized mortality rate, TIGER/Line, Topologically Integrated Geographic Encoding and referencing.


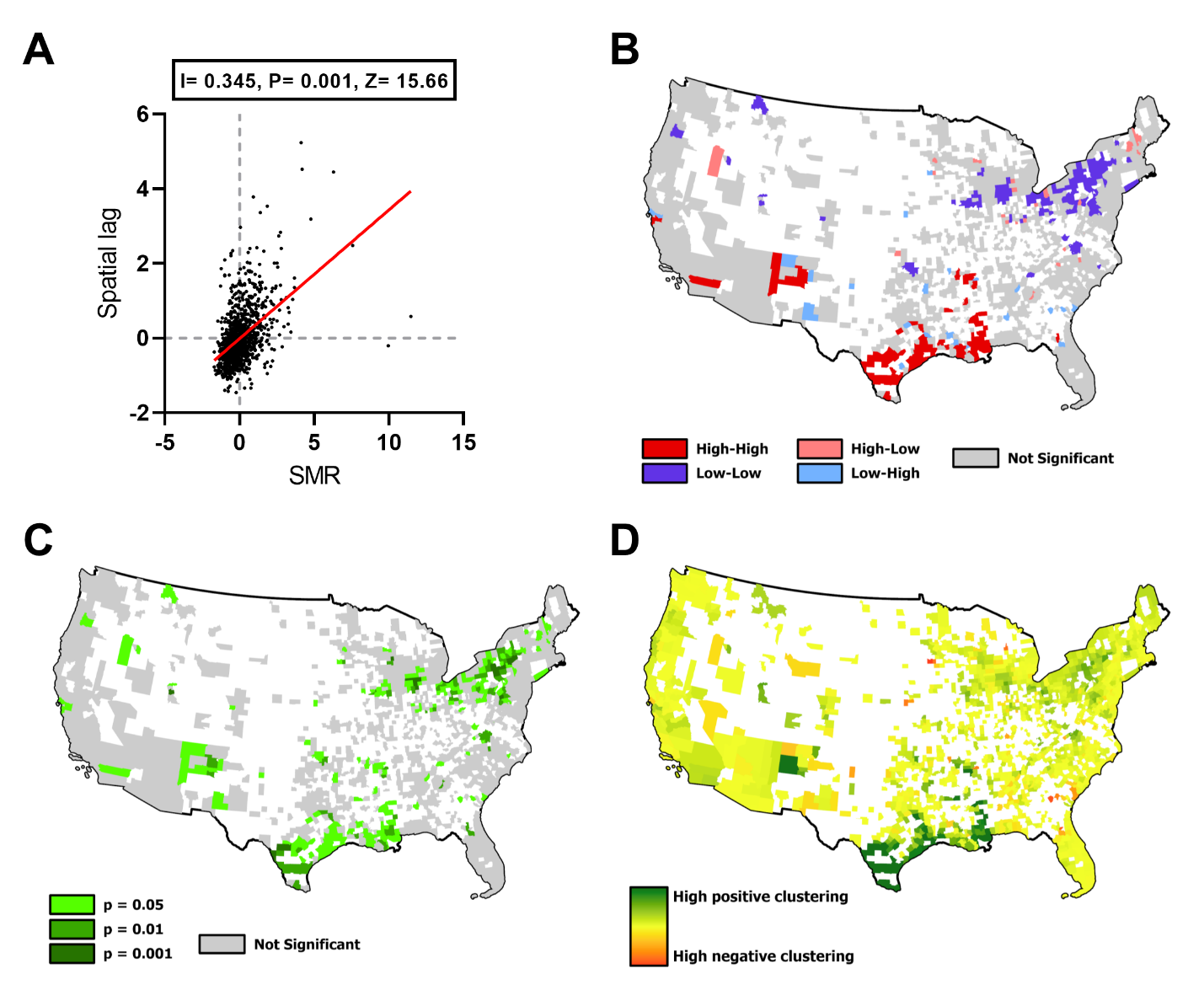


**Fig. S2: Geospatial analysis of mortality from primary liver cancer (2000-2010).** (A) Global spatial association analysis of standardized mortality rate (SMR) in 2000-2010 with global Moran’s I indicated. (B) Local spatial association and clustering based on local Moran’s I-statistic. (C,D) Local indicators of spatial association analysis with (C) significant clusters shown in color, and with non-significant changes in gray and (D) associated p values.

**
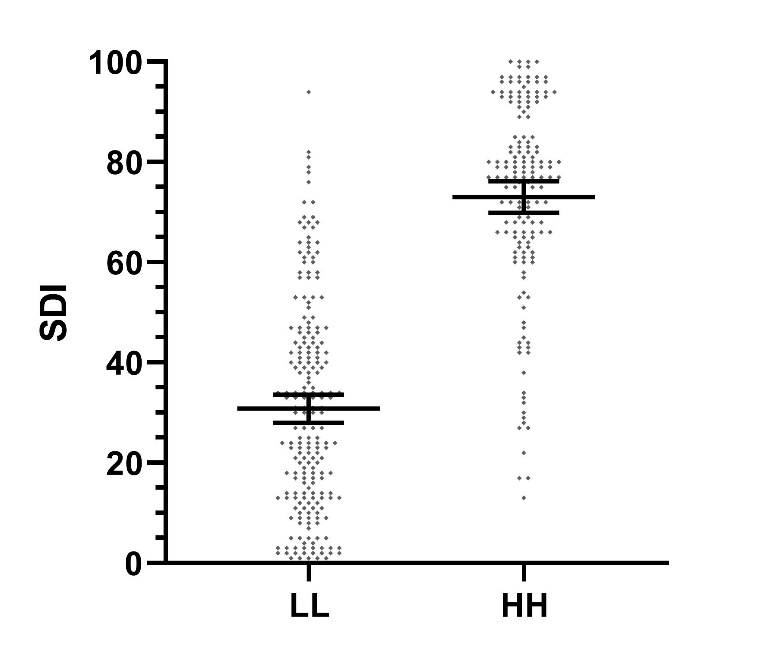
**

**Fig. S3:** **Social deprivation index within clusters of liver cancer mortality.** Gray dots within each column represent the social deprivation index (SDI) of counties classified as hot-spots (HH) or cold-spots (LL) in 2010-2020. The mean value and 95% confidence intervals are shown for each.

**Table S1: Data sources.**

| **Source** |  | **Variables** | **Time frame** |
| --- | --- | --- | --- |
| U.S. Census Bureau; 5-year American Community Survey | <https://data.census.gov/>.  Retrieved May 25th, 2023 | Males per 100 females, Median age (years), % pop. >65 years of age, % pop. that is of any minority, Ratio of black to white, % pop. that is Hispanic, % pop. that is Asian, Total pop., | 2005-2010, 2015-2020 |
|  |  | % pop. unemployed, Per capita income (U.S. dollars), Median household income (U.S. dollars), |  |
|  |  | % pop. >25 years of age with bachelors, % pop. >25 years of age with high school equivalency |  |
|  |  | Household size (persons), Family size (persons), Single parent households, % pop. living in mobile homes, % pop. living in group homes, % households with more people than rooms, % households without car, % pop. who speaks English less than very well |  |
|  |  | % pop. with disability, % pop. >65 years of age with disability | 2015-2020 |
| United States Department of Agriculture Food Environment Atlas | <https://www.ers.usda.gov/data-products/food-environment-atlas/data-access-and-documentation-downloads/>.  Retrieved May 25th, 2023 | % pop. with low food access | 2010, 2015 |
|  |  | % pop. using the supplemental nutrition assistance program | 2012, 2017 |
| U.S. Census Bureau; Decennial survey | <https://data.census.gov/>  Retrieved May 25th, 2023 | Percentage of households in rural areas | 2010, 2020 |
| U.S. Census Bureau Small Area Income and Poverty Estimates (SAIPE) Program | <https://www.census.gov/programs-surveys/saipe.html>  Retrieved June 14th, 2023 | % pop. without health insurance |  |
| U.S. Census Bureau Small Area Health Insurance Estimates | <https://www.census.gov/programs-surveys/sahie.html>.  Retrieved June 14th, 2023 | % pop. in poverty |  |
| Center for Disease Control: PLACES database | <https://www.cdc.gov/places/index.html>.  Retrieved June 13th, 2023 | % pop. that has routine yearly checkups, >18 years of age receiving cholesterol screening, with self-rated poor physical health for >14 days, with self-rated poor mental health for >14 days, reporting binge drinking, with hypertension, taking meds for hypertension | 2019-2020 |
| Center for Disease Control Diabetes Surveillance Network | <https://gis.cdc.gov/grasp/diabetes/DiabetesAtlas.html>.  Retrieved May 31st, 2023 | % pop. with diabetes, obesity, primary care physicians | 2018 |
|  |  | Food environment index | 2018 |
| 2022 TIGER/LINE shapefile ^a^ | <https://www.census.gov/geographies/mapping-files/time-series/geo/tiger-line-file.2022.html#list-tab-790442341>.  Retrieved May 19th, 2023 | County size (meters^2^) | 2022 |
| Homeland Infrastructure Foundation-Level Data: Hospitals shapefile ^b^ | <https://hifld-geoplatform.opendata.arcgis.com/datasets/75079bdea94743bcaca7b6e833692639_0/explore>.  Retrieved June 2nd, 2023 | Total hospitals, hospital beds, General medical/surgical hospitals, General medical/surgical hospital beds, Specialty hospitals, Specialty hospital beds | 2023 |

a. County size from 2022 TIGER/LINE shapefile and total county population from American Community Survey was used to calculate population density (population divided by sq. mile). b. Open hospitals within each county were summed, divided by the total county population obtained from American Community Survey, and then multiplied by 100,000. Abbreviations: pop., population.

**Table S2: Health and healthcare in counties that remained non-hot-spots or became new hot-spots.**

^a^ per 100,000 population, ^b^ including for psychiatric/substance abuse, **^c^** Excluding psychiatric/substance abuse, ^d^ mean during 2010-2020. Pop: population

| **Variable** | **Remained Non hot-spots** ^d^ | **New hot-spots** ^d^ | ***p*-value** |
| --- | --- | --- | --- |
| % pop. with low food access | 20.05 | 24.08 | 0.018 |
| % pop. with diabetes | 9.24 | 9.59 | 0.2 |
| % pop. with obesity | 29.85 | 29.43 | 0.6 |
| % pop. with hypertension | 35.32 | 37.49 | 0.025 |
| % pop. taking meds for hypertension | 76.38 | 75.78 | 0.3 |
| % pop. with disability | 14.89 | 16.62 | 0.010 |
| % pop. >65 years of age with disability | 35.41 | 40.41 | <0.0001 |
| % pop. with self-rated poor physical health for >14 days | 11.31 | 12.63 | <0.0001 |
| % pop. with self-rated poor mental health for >14 days | 14.76 | 16.03 | <0.0001 |
| % pop. reporting binge drinking | 15.87 | 15.76 | 0.8 |
| Primary care physicians ^a^ | 64.90 | 60.85 | 0.4 |
| % pop. that has routine yearly checkups | 75.86 | 74.96 | 0.2 |
| % pop. >18 years of age receiving cholesterol screening | 84.37 | 84.62 | 0.6 |
| Total hospitals ^a, b^ | 2.82 | 4.94 | <0.0001 |
| Total hospital beds ^a^ | 312.4 | 416.1 | 0.018 |
| General medical/surgical hospitals ^a,^ ^b^ | 2.24 | 3.13 | 0.0009 |
| General medical/surgical hospital beds ^a^ | 272.10 | 328.50 | 0.1 |
| Specialty hospitals ^a, c^ | 0.09 | 0.31 | 0.018 |
| Specialty hospital beds ^a^ | 4.10 | 23.61 | 0.017 |

**Table S3: Features of hot-spot counties that became not hot-spots.** Variables relating to income and food access were compared in 17 counties identified as hot-spots for liver cancer mortality in 2000-2010 became non-hot-spots in 2010-2020.

| **Variable** | **2000-2010**  **average** | **2010-2020**  **average** | **p-value** |
| --- | --- | --- | --- |
| Per capita income  (U.S. dollars) | 23,494 | 29,441 | 0.063 |
| Median household income  (U.S. dollars) | 49,378 | 59,816 | 0.1 |
| % population without health insurance | 26.55 | 18.28 | <0.0001 |
| % pop. poverty | 18.15 | 15.14 | 0.2 |
| % population unemployed | 8.44 | 5.72 | 0.009 |
| % population using the supplemental nutrition assistance program | 17.14 | 14.83 | 0.051 |
| % population with low food access | 24.22 | 23.28 | 0.9 |
